# Construct Validity of a Virtual Reality Simulator for Surgical Training in Knee Arthroscopy

**DOI:** 10.7759/cureus.15237

**Published:** 2021-05-25

**Authors:** Miguel J Palet, Marcela Antúnez-Riveros, Maximiliano Barahona

**Affiliations:** 1 Department of Orthopedic Surgery, Faculty of Medicine, University of Chile, Santiago, CHL; 2 Department of Health Sciences Education, Faculty of Medicine, University of Chile, Santiago, CHL

**Keywords:** arthroscopy, knee, simulation training, surgical simulation, virtual reality

## Abstract

Objective

Surgical techniques are learned gradually throughout an orthopedic residency. Training on real patients carries drawbacks such as limited access and elevated risk. Alternatively, surgical simulation allows residents to practice in a safe environment with greater access to standardized surgical tasks. Virtual reality simulators display images inside an artificial joint, often providing real-time haptic feedback to allow for realistic interaction. The objective of this study was to evaluate the construct validity of a virtual reality simulator for knee arthroscopy by analyzing the capacity of system parameters to distinguish between expert and novice surgeons.

Design

This comparative cross-sectional study contrasts the automated performance reports for novice and expert orthopedic surgeons after executing surgical tasks on the ARTHRO Mentor virtual reality simulator.

Setting

Surgical simulation center at the University of Chile Clinical Hospital, Santiago, Chile.

Participants

The novice group consisted of 20 second-year orthopedic and traumatology residents at the University of Chile School of Medicine. The expert group consisted of 10 experienced arthroscopic surgeons. All participants carried out standardized tasks in the knee arthroscopy virtual reality simulator. The median performance scores of the two groups were compared, and multivariate logistic regression was performed to assess the capacity of the system to discriminate between the two groups.

Results

Median performance on the vast majority of surgical tasks was superior for the expert group. The expert group had performance values equal to or higher than the novice group on 43 of the 44 variables recorded for the basic tasks and 74 of the 75 advanced task variables. The multivariate logistic regression analysis discriminated expert from novice users with 100% accuracy.

Conclusion

The virtual reality simulator for knee arthroscopy showed good construct validity, with performance metrics accurately discriminating between expert and novice users.

## Introduction

Arthroscopy has become the gold standard for treating joint pathology. Knee arthroscopy is the most commonly performed traumatological procedure in the United States [1] and, likewise, at the University of Chile Clinical Hospital where this study took place. Surgery has classically been taught in the operating room by an expert, using real patients [2-4], with all of the difficulties that this approach involves. Drawbacks of training with real patients include high cost [4] and, more significantly, increased risk for patients [5]. Given that medical errors are the third-leading cause of death in the United States [6], this risk is no longer tolerable. Current surgical procedures require a learning curve to minimize errors that can be more frequent and severe in less experienced hands [7]. Practical training opportunities may be limited for orthopedic and traumatology students due to patient’s safety, along with regulations reducing the weekly schedule for residents to 80 hours [8]. To improve the quality of surgical training, therefore, programs have introduced simulation training, including the use of bench models, live animals, cadavers, high fidelity simulators, and virtual reality surgical simulators [3,9]. The basic concept of these approaches is that the first stages of surgical training can take place outside the operating room, with residents proceeding to train on real patients after having achieved a skill level equivalent to the automation stage of learning in the Fitts and Posner model [10], performing tasks with speed and precision [11] or the competent stage of the Dreyfus model [12,13]. Virtual reality simulations, based on the technology pioneered in aviation simulation [14], make use of computerized phantom extremities displaying the inside of a joint on a screen. The student performs procedures inside the phantom joint using system-linked instruments that provide instantaneous haptic feedback. This training qualifies as deliberative practice [15], as it presents short tasks to motivated subjects, offering immediate feedback and the opportunity for multiple repetitions. Preliminary evidence suggests that this teaching method is useful; for instance, virtual reality simulation has been shown to reduce operating times in laparoscopic surgery [16]. In addition to its utility as a teaching tool, simulation provides a way to evaluate performance. Considering the Miller pyramid for assessing clinical skills, surgical simulation corresponds to the third level of competence, or demonstration of learning [17]. In the Miller model, the third and fourth levels (“shows how” and “does”) represent the behavioral levels of competence. Virtual reality simulators provide an automated numerical report after each task, supplying an objective measure of the performance [18].

The validity of simulated training can be assessed along five dimensions [19-21]. Face validity is the subjective degree to which the model resembles the real surgical situation. Content validity reflects the extent to which the model covers the relevant aspects of the real situation. Both of these types of validity can be measured using surveys. Construct validity is the capacity of the system to effectively simulate the relevant skill and to discriminate between expert and novice performance [20,22,23]. Concurrent validity measures agreement between the simulator and another type of previously validated assessment. Finally, predictive validity is the capacity of the simulator to predict the clinical performance of subjects exposed to the training model, measuring the transfer of skills to the real surgical environment [2,21,24,25].

In Chile, there has been no reported evidence to date regarding the use of virtual reality simulators in traumatology programs, although surgical simulation has been studied [26] and validated [27] in the context of laparoscopic surgery.

The objective of this study was to evaluate the construct validity of a knee arthroscopy virtual reality simulator used in an orthopedic surgeon residency program.

## Materials and methods

Study design

A comparative cross-sectional study was conducted to evaluate the automated performance reports produced by the ARTHRO Mentor™ knee arthroscopy simulator (SimbionixTM, Cleveland, OH, USA), comparing the performance of novice and expert orthopedic surgeons.

Participants

All 20 second-year residents in the traumatology and orthopedic program at the University of Chile School of Medicine were enrolled in the study and assigned to the “novice” group. The expert group was made up of 10 attending traumatologists from the same department with experience in arthroscopy. The inclusion criteria for the expert group were: formal academic education in arthroscopy, surgical arthroscopy, or sports traumatology; at least three years of experience in arthroscopic surgery; and at least 500 arthroscopic procedures completed [19]. Sample size calculations were not performed and the participant number was defined according to their availability; in the case of the students, all second-year residents were included, and in the case of the experts, all participants who met inclusion criteria and agreed to participate were enrolled.

Definition of tasks

**Figure 1:**
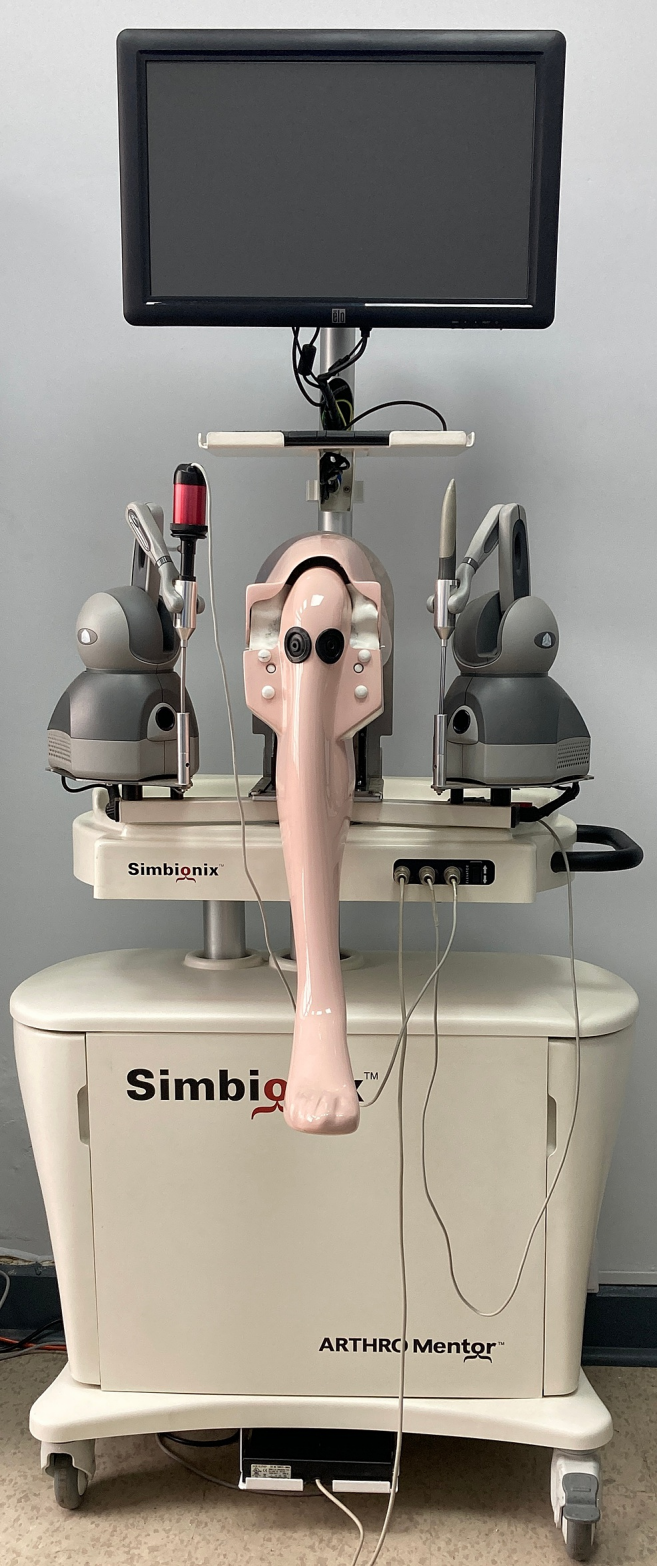
ARTHRO Mentor™ knee arthroscopy simulator

The surgical tasks were defined as the standardized modules for the Simbionix™ ARTHRO Mentor™ knee arthroscopy simulator (Figure 1).

The basic tasks were defined as the 11 exercises in the FAST (Fundamentals of Arthroscopic Surgery Training) course included in the simulator software. These tasks were: steadiness of the camera and arthroscope, image orientation, image centering, telescoping, deliberate linear scope motion, periscoping, tracking a moving target with the scope, basic probe triangulation, touch and probe of a stationary target, simultaneous image tracking and probing of a moving target, and measurement of articular dimensions with the tip of a probe.

The seven advanced techniques were: arthroscopic visual examination, diagnostic arthroscopy with advance probe examination, diagnostic arthroscopy of a random intra-articular pathology, meniscectomy of a radial lateral tear, loose body removal, femoral condyle repair with microfractures, and tunnel placement for anterior cruciate ligament (ACL) reconstruction.

The simulator records a series of performance parameters for each task. Most of the exercises include the following parameters: completion time, the accuracy of camera and instrument use, percentage time in partial and perfect alignment, arthroscope path distance, and camera path distance. For the advanced procedures, additional safety parameters are included: camera collisions with the tissue and capsule, blind use of instruments, and iatrogenic chondral damage.

All procedures were carried out at the surgical simulation centers in the University of Chile Clinical Hospital, under the direction of the principal investigator.

All novices and experts (n=30) performed 18 tasks. The students received instantaneous feedback from an expert after finishing each task and were given the opportunity to repeat the tasks using the suggestions and corrections provided. However, only the scores for the first attempt of both groups were used for the study analysis. The performance data generated automatically by the simulator software for the first attempt at each task by each subject were recorded; no performance thresholds were applied. The simulator recorded a total of 238 variables for the 18 tasks for each subject. Some variables were fixed rather than varying according to performance. As these variables were identical for all participants, they were not analyzed. Finally, the remaining 119 metrics from the 18 tasks were saved in an Excel (Microsoft) spreadsheet for a total of 30 subjects, 20 novices, and 10 experts.

Statistical analysis

Descriptive and inferential statistics were performed using Stata 15 software (StataCorp CP, College Station, TX, USA). Considering the data distribution, nonparametric analyses were performed, Wilcoxon nonparametric medians difference test was used for unpaired samples to compare the median scores between groups [28]. A multivariate logistic regression analysis was also performed using a priori probability information [29]. Statistical significance was defined as p<0.05 for differences between groups.

## Results

The group of 20 novices included all of the second-year residents in the program. None of the residents had previous experience with surgical simulators or surgical arthroscopy. The age range was 26 to 39 years, and all were male. All 10 experts were surgeons experienced in knee arthroscopy; three also had experience with cadaveric arthroscopy and three with shoulder arthroscopy. The age range was 38 to 59 years, and all were male.

The experts had superior performance results for the vast majority of tasks. A total of 44 variables were recorded for the 11 tasks in the basic FAST course. The median expert score was equal to or higher than the median novice score on 43 (98%) of these variables. The novice score was higher on only one variable, the number of times that probe was out of contact, in task 10, but this difference was not significant. The detailed results for the FAST tasks were as follows:

Basic task 1, steadiness of the camera and arthroscope: Both novices and experts demonstrated perfect accuracy and alignment. 

Basic task 2, image orientation: Accuracy was worse for novices than experts. The percentage of time in perfect alignment was worse for novices than experts. The efficiency of rotational movement was worse for novices than experts.

Basic task 3, image centering: Accuracy was significantly higher among experts than novices (p=0.0414). The experts were also faster than the novices (p=0.0399). The efficiency of movement metrics was also higher for the experts than the novices (p=0.0248) percentage of time in perfect alignment was also better in the expert group but this difference was not significant.

Basic task 4, telescoping: Time in perfect alignment was significantly higher for the expert group (p=0.0075). The percentage of time in perfect alignment was worse for novices than experts, but this difference was not significant.

Basic task 5, deliberate linear scope motion: The performance was superior for the expert vs. novice group for all four variables, but only in three the difference was significant. Time in perfect alignment (p=0.0206), efficiency of linear movement (p=0.0094), and total time (p=0.0036).

Basic task 6, periscoping: The performance was significantly superior for the expert vs. novice group for all five variables: accuracy, time in perfect alignment, periscope efficiency, camera movement, and total time.

Basic task 7, tracking a moving target with the scope: Differences between groups were not statistically significant.

Basic task 8, basic probe triangulation: The expert group had significantly superior performance on three of four variables: probe accuracy (p=0.0082), time in perfect alignment (p=0.023), and total time (p=0.0002).

Basic task 9, touch and probe of a stationary target: The expert performance was superior to novice performance for all three variables, but the only statistically significant difference was for total time (p=0.0093).

Basic task 10, simultaneous image tracking and probing of a moving target: The expert performance was superior for two of three variables. The number of times that probe was out of contact was the only variable that novices had superior performance than experts. The differences were not statistically significant for the three variables.

Basic task 11, measurement of articular dimensions with the tip of a probe: The expert performance was superior to novice performance for all three variables, with statistically significant differences for two: efficiency of measurement (p=0.0012) and total time (p=0.0073).

Table 1 compares expert with novice performance on the FAST course tasks, showing 35 of the 44 variables recorded. The remaining 11 variables were percentages of time in partial alignment, which corresponded to a value of 100% minus the given value for time in perfect alignment.

**Table 1:** Comparison of novice and expert performance in basic FAST task metrics Time is in seconds, distances in centimeter, attempts in the number of times. Other scores are calculated as a percentage. FAST: Fundamentals of Arthroscopic Surgery Training

Task		Median novice score	Median expert score	Statistical significance (p-value)
FAST 1	Percentage of accuracy	100	100	0.2398
	Percentage of time in perfect alignment	100	100	0.2277
FAST 2	Percentage of accuracy	50	66	0.0923
	Percentage of time in perfect alignment	97.5	99	0.5156
	Efficiency of rotational movement	50.7	65	0.078
FAST 3	Percentage of accuracy	63	73.5	0.0414
	Percentage of time in perfect alignment	90.5	94	0.1514
	Efficiency of movements	64	88	0.0248
	Total time	45	37.5	0.0399
FAST 4	Percentage of time out of alignment	2.5	1.5	0.273
	Percentage of time in perfect alignment	95	98	0.0075
FAST 5	Percentage of accuracy	70.5	85	0.097
	Percentage of time in perfect alignment	75	83	0.0206
	Efficiency of linear movement	43	73	0.0094
	Total time	28.5	26.5	0.0036
FAST 6	Percentage of accuracy	28.5	45.5	0.0243
	Percentage of time in perfect alignment	58.5	71	0.0262
	Periscope efficiency	39	67	0.0008
	Camera movement	108	18.5	0.00001
	Total time	269.5	110	0.00001
FAST 7	Percentage of time out of alignment	0	0	0.1201
	Percentage of time in perfect alignment	97,5	98	0.2257
FAST 8	Percentage of probe accuracy	50	66	0.0082
	Percentage of time in perfect alignment	82.5	87	0.023
	Probe efficiency	81.5	87	0.659
	Total time	145	60	0.0002
FAST 9	Percentage of probe accuracy	54	60	0.8766
	Probe efficiency	100	100	0.5639
	Total time	49.5	39.5	0.0093
FAST 10	Number of times that probe was out of contact	4,5	5.5	0.8418
	Percentage of time in perfect alignment	93	94.5	0.4145
	Efficiency of probe movement	35.5	40	0.8258
FAST 11	Attempts before successful task completion	2	1.5	0.3477
	Efficiency of measurement	16	52.5	0.0012
	Total accumulated time	191.5	69.5	0.0073

**Figure 2:**
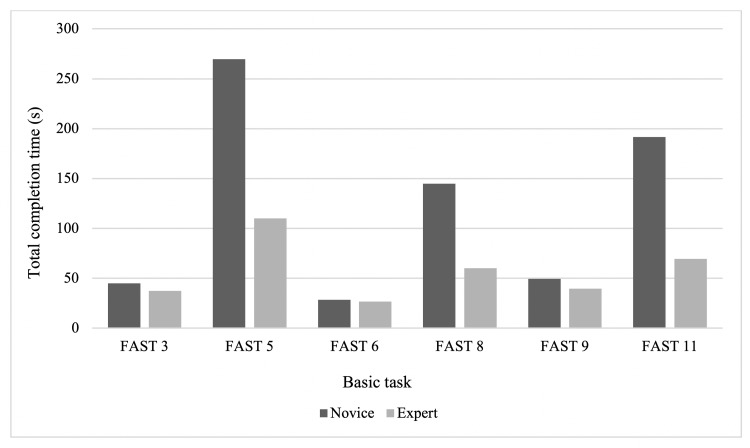
Median completion time for basic tasks Comparison of the time required for novices vs. experts to complete each basic FAST task for which this variable was recorded. Results are expressed in seconds. All differences were statistically significant (p<0.05).

The experts performed the six timed basic tasks significantly faster than the novice surgeons, all with statistical significance (p<0.05) (Figure 2). The expert performance was significantly superior to novice performance for 22 of the 44 variables in the basic tasks (50%).

A total of 75 variables were recorded for advanced tasks. The performance of the experts was superior to that of the novices for the vast majority of these tasks.

Advanced task 1, arthroscopic visual examination: The performance of the expert group was superior for all six variables, and five of these differences were statistically significant: total time, total camera distance, number of collisions with the capsule, number of camera-tissue collisions, and the average time to locate a target. The experts also showed superior performance for the percentage of camera steadiness, but this difference was not significant (p=0.3099).

Advanced task 2, diagnostic arthroscopy with advance probe examination: Notably, expert performance values were higher than novice values for all 11 variables, and 10 of these differences were statistically significant. The median completion time for the expert group was about half that of the novice time (170.5 vs. 346 s) (p<0.00001). Furthermore, total camera and probe distances were approximately half the novice distances (47 vs. 123 and 103.5 vs. 208 cm, respectively) (p=0.0003 and p=0.0056). Finally, the experts had fewer camera-tissue collisions (p=0.0001) or collisions with the capsule (p=0.023) and fewer instances of blind probing (p=0.0005).

Advanced task 3, diagnostic arthroscopy of a random intra-articular pathology: The expert performance was superior to novice performance for all eight variables, and five of the differences were statistically significant. Completion time was markedly faster for the expert vs. novice group (p<0.00001), and the experts did not commit any errors in identifying intra-articular pathologies nor did they omit any pathologies. The novices, in contrast, only had 65% (13/20) accuracy in identifying pathologies and omitted 35% (7/20) of pathologies.

Advanced task 4, meniscectomy of a radial lateral tear: For this task, the participants were asked to perform a partial meniscectomy for a radial lesion of the lateral meniscus. A total of 22 variables were recorded. Performance values were significantly higher for the expert group on half of these variables. Notably, in terms of safety, the experts did not cause any cartilage damage, unlike the novices (0 vs. 58 mm^2^ of area damaged) (p=0.0392) and had fewer camera-tissue collisions (7.5 vs. 16) (p=0.001).

The system provided an automatic overall score for advanced tasks 5 to 7, from 0 to 10.

Advanced task 5, loose body removal: five of the nine metrics was statistically significant. The overall scores for the expert group were significantly higher than those of the novices (7.2 vs. 4.7) (p=0.0004). Completion time was again markedly faster for the expert group (64 vs. 151.5 s) (p=0.0001), and the camera and instrument path lengths were notably shorter for the expert vs. novice group.

Advanced task 6, femoral condyle repair with microfractures: The median expert score was equal or higher than that of the novices for 10 of the 11 variables recorded. The novice values were higher for one variable, but this difference was not statistically significant. While overall scores were similar (7.5 vs. 7.2 for the experts vs. novices), the completion time was markedly faster for the experts (143 vs. 260.5 s) (p=0.0003).

Advanced task 7, tunnel placement for ACL reconstruction with single-band technique: The expert performance was superior to that of novices for all eight variables recorded, and the differences were statistically significant for seven metrics. Notably, the completion time was faster for the expert vs. novice group (99.5 vs. 139.5 s) (p=0.0003), and the overall score was higher (8.6 vs. 6.1) (p<0.00001).

The data for the advanced tasks and the statistical significance are shown in Table 2.

**Table 2:** Comparison of novice and expert performance scores on advanced tasks Efficiencies are expressed in percentage, time in seconds, distance in centimeter (unless mm is specified), and roughness in Newton. The overall score is calculated automatically by the simulator software, with a range of 0 to 10. Other scores are calculated as a percentage. NA: Not available;  ACL: anterior cruciate ligament

Task		Median novice score	Median expert score	Statistical significance (p-value)
1. Arthroscopic visual examination	Total time	191	114	0.0007
	Total camera distance	113.5	71	0.0045
	Number of collisions with the capsule	14	9.5	0.0103
	Number of camera- tissue collisions	68.5	45	0.0037
	Percentage of camera steadiness	61	69	0.3099
	Average time to locate a target	19	11	0.0001
2. Diagnostic arthroscopy with advance probe examination	Total time	346	170.5	0.0001
	Total camera distance	123	47	0.0003
	Total tool distance	208	103.5	0.0056
	Number of camera-tissue collisions	16	7	0.0001
	Total time of camera-tissue collisions	105	53	0.0073
	Total time of blind tissue probing	20	4	0.0005
	Number of times irrelevant organs were touched	35	19.5	0.0015
	Total distance tools were moved blindly	92.5	33	0.0006
	Number of collisions with the capsule	8	4	23
	Percentage of probe steadiness	19.5	20.5	0.8087
	Average time to probe a target	38.5	18.5	<0.00001
3. Diagnostic arthroscopy of a random intra-articular pathology	Total time	262	73.5	<0.00001
	Total camera distance	130	44.5	<0.00001
	Number of camera- tissue collisions	17	5.5	<0.00001
	Total time of camera- tissue collisions	78	17	0.0002
	Number of collisions with the capsule	0	0	0.1364
	Identification of correct pathology	13/20	10/10	NA
	Omission of intra-articular pathology	7/20	0/10	NA
	Percentage of the articular area examined	80	90	0.0062
4. Meniscectomy of a radial lateral tear	Total time	355.5	183.5	0.0004
	Total camera distance	50.5	17.5	0.0002
	Total tool distance	165.5	97.5	0.0366
	Number of camera- tissue collisions	16	7.5	1
	Total time of camera- tissue collisions	150.5	50	0.0155
	Total distance tools were moved blindly	71	37.5	0.0248
	Total time tools were moved blindly	53.5	30	0.0064
	Number of collisions with the capsule	2	2	0.5011
	Diagnostic time	63	52.5	0.1591
	Percentage of palpated tear	18.5	61	0.2984
	Time punch was used	118.5	64	0.0146
	Punch movement	67.5	39.5	202
	Number of blind punching	2	1	0.1178
	Number of times an irrelevant organ was touched	22	16	0.1587
	Distance of open punch movement	61.5	28.5	0.0677
	Percentage of remaining meniscus	96	96	0.6395
	Punching efficiency	59	61	0.5667
	Time shaver was used	90	37	<0.00001
	Shaver movement	72.5	23.5	0.0013
	Total time of blind shaving	0	0	0.4622
	Area of cartilage damage (mm^2^)	58	0	0.0392
	Shaving efficiency	48	70	0.0501
5. Loose body extraction	Overall score	4.7	7.2	0.0004
	Unsuccessful grasping attempts	10.5	2	0.0157
	Covered distance: camera	45.9	13.55	0.0006
	Covered distance: grasper	87.4	35.65	0.0004
	Covered distance: open grasper	79.2	14.35	<0.00001
	Roughness: camera	0	0	0.1838
	Roughness: grasper	12.5	12	0.2456
	Roughness: open grasper	12.5	12	0.0521
	Total time	151.5	64	0.0001
6. Femoral condyle repair with microfractures	Overall score	7.2	7.5	0.0502
	Percentage of damaged surface covered	85	81	0.1517
	Percentage of effective microfractures	100	100	0.2594
	Maximum depth of microfractures (mm.)	4	4	0.3517
	Minimum distance between microfractures (mm.)	1	2	0.2355
	Number of microfractures in healthy area	0	0	0.2841
	Covered distance: awl	99.9	41.65	0.0001
	Covered distance: camera	57.05	25.5	0.0094
	Roughness: awl	0	0	0.6711
	Roughness: camera	0	0	0.4303
	Total time	260.5	143	0.0003
7. Tunnel placement for ACL reconstruction	Overall score	6.1	8.6	<0.00001
	Covered distance: camera	82.05	24.35	0.0004
	Covered distance: probe	107.9	34.3	0.0004
	Roughness: camera	1	0	0.0336
	Roughness: probe	5	4	0.1953
	Femoral tunnel distance (mm.)	7	3	0.0095
	Tibial tunnel distance (mm.)	5	1.5	0.0079
	Total time	239.5	99.5	0.0003

The expert group had superior performance on 65 of the 75 variables analyzed for the seven advanced tasks (87%). Most of these differences were statistically significant (48/75; 64%). The performance was similar between groups for nine tasks. The novice group had superior performance on only one variable (1.3%), in the microfractures procedure task: percentage of damaged surface covered. This difference was not statistically significant (p=0.1517). Table 3 provides a summary of the performance metrics for advanced tasks.

**Table 3:** Summary of all 75 variables for the seven advanced tasks, comparing the performance of expert and novice surgeons ACL: anterior cruciate ligament

Task	Experts had superior performance	Similar performance	Novices had superior performance	Total
1 Arthroscopic visual examination	6	0	0	6
2 Diagnostic arthroscopy with advance probe palpation	11	0	0	11
3 Diagnostic arthroscopy of random intra-articular pathology	6	0	0	6
4 Meniscectomy of a radial lateral tear	21	3	0	24
5 Loose body extraction	8	1	0	9
6 Femoral condyle repair with microfractures	5	5	1	11
7 Tunnel placement for ACL reconstruction	8	0	0	8
Total	65	9	1	75

**Figure 3:**
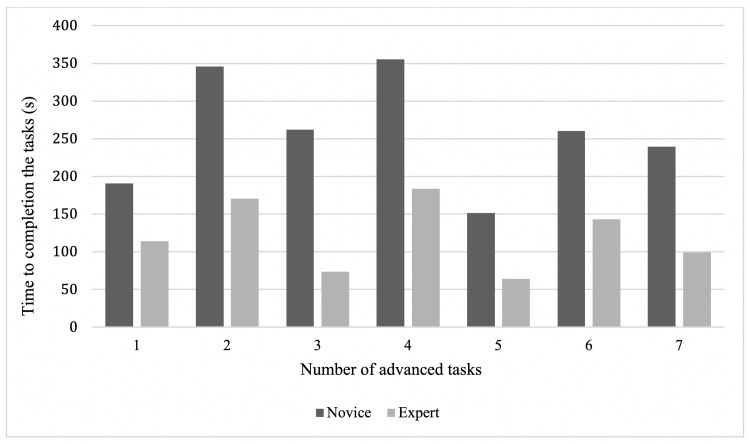
Median completion time for advanced tasks Median completion time for advanced tasks, comparing novice and expert surgeons. Time is expressed in seconds. The differences for all tasks were statistically significant (p<0.05).

The experts had a statistically significant faster completion time for all seven advanced tasks. Expert completion times were roughly half those of the novices for four tasks (Figure 3).

Multivariate logistic regression was performed using a priori probability ratios. A total of 27 variables were statistically defined as the most representative of performance differences between the two groups. Based on the scores for these variables, the model then predicted the group into which each participant would be classified. The model classified all of the students as novices and all of the experienced surgeons as experts, as shown in Table 4. In other words, the error rate was zero for both groups. Classification accuracy remained without errors even when the analysis was performed without prior information about the distribution; that is when the performance of the 30 subjects was analyzed with a 50% probability of belonging to either group, not the 33% and 66% a priori probability ratio used before.

**Table 4:** Multivariate logistic regression The multivariate logistic regression used the performance variables provided by the virtual reality surgical simulator to predict the true role of each surgeon with 100% accuracy.

True role	Classified as Novice	Classified as Expert
Novice	100%	0%
Expert	0%	100%

## Discussion

The performance of the expert group was superior to that of the novices for the vast majority of the variables analyzed, and these differences were largely significant. The difference between expert and novice scores were statistically significant for 50% of the basic and 64% of the advanced task variables. The differences were most pronounced for advanced tasks that required the surgeons to use expert judgment and fluidly integrate various motor skills to evaluate or treat a simulated pathology, rather than to merely demonstrate a single motor skill in isolation. This level of mastery represents the most advanced level in the Fitts and Posner model, that is, the autonomous stage of learning [11], and at least the proficient nor the expert stage of Dreyfus [12,13]. This result is logical given that the advanced tasks required the students to complete surgeries that they had not previously performed, while the basic tasks did not demand such a high level of dexterity. Furthermore, the parameters for the basic tasks do not have a direct clinical correlation, unlike the advanced tasks that are quite similar to real surgical procedures. Therefore, the skill of the expert surgeons was better demonstrated in these advanced tasks, reflected in faster completion times and more skillful handling of the instruments. Specifically, the expert scores reflected shorter punch, camera, and probe paths and fewer errors that would be likely to produce intra-articular damage, such as camera collisions with tissue or palpation of irrelevant structures. Expert scores were also superior in terms of roughness of instrument use, steadiness, blind use of instruments, and efficiency of shaver use, resulting in less cartilage damage. Finally, from a safety standpoint, the novice group committed more errors than expert practitioners; the hope is that the students would learn from the feedback and avoid these errors when working with real patients.

To assess construct validity defined as the capacity of the system to effectively measure the ability simulated, we assessed the ability of the performance metrics to discriminate between expert and novice surgeons [21]. As a first step, we compared the median performance scores of the two groups. These results were statistically significant; however, the preferred method for assessing the validity of a surgical simulator is multivariate logistic regression using a priori probability ratios, which was performed as the second step in our study. This analysis demonstrated 100% accuracy in classifying the participants as novices or experts, providing a robust validation of the simulator. The analysis was first conducted using the a priori probabilities of 33% and 66% for the expert and novice categories, respectively, as the study sample included 10 experts and 20 novices. However, the classification remained 100% accurate when a priori probabilities of 50% were applied, offering an even stronger validation of the discrimination capacity of the simulator.

As an advantage, the virtual reality arthroscopy simulator can be used as a reliable and objective tool to evaluate the acquisition of surgical competencies. To pass the simulated tasks [18], the resident must provide a practical demonstration of the skill, effectively performing the task in a simulated manner, corresponding to the third level in the Miller model for assessing competence [17]. This learning model “shows how” corresponds to the evaluation of a performed skill rather than knowledge. Miller theorized four levels of skill or clinical competency acquisition, ordered from least to greatest complexity. The first two levels of the pyramid correspond to the demonstration of acquired knowledge, which may be memorized but not yet applied. The first level is “knowing,” that is, having knowledge about the procedure, followed by “knowing how to,” in which the subject is capable of describing how to perform a task but may not be able to practically demonstrate the skill. The final two levels both require a practical demonstration of the skill. At the third level, “shows how,” subjects can demonstrate the task. Progressing towards this level of learning is the area in which the simulation system is most useful, as it allows subjects to perform tasks that they had previously studied in order to acquire and demonstrate a skill or competency. Finally, the peak of the pyramid is “doing,” which in this context means demonstrating the skill in real patients. Formal practical assessments are rarely used in surgical training programs. The practical evaluations used are generally subjective, with the teaching surgeon assessing and finally approving the performance of his or her students in the operating room. Surgical simulators provide a more objective evaluation of learned skills. This approach may also be useful in sports medicine training and in the certification of orthopedic surgeons.

We had a positive impression practicing with the virtual reality simulator, although the initial cost can be high, the maintenance of the equipment was not. We highlight, as well as previous studies [4], the great availability, transversality, and the opportunity to practice at any time without the need for expert supervision.

A strength of this study is a larger sample as compared to previous research on this topic [22,23,28,29]. In prior virtual reality simulation studies, the samples of novices were often large, but the number of experts evaluated has tended to be low. Another strength is the depth of performance variables analyzed. While previous research has tended to focus on completion time, this study analyzed 119 variables separately and included 27 variables in the multivariate analysis, providing more support for the construct validity of the tool.

One limitation of this study was the lack of clinical outcomes correlation. It would be interesting to evaluate the transfer of learning to the operating room or to assess the performance of residents who complete virtual reality simulation training. The literature is limited in these types of studies; our intention is to continue in that way.

## Conclusions

The ARTHRO Mentor™ knee arthroscopy simulator demonstrates construct validity, accurately discriminating novice from expert surgeons.

Virtual reality simulation to teach surgical skills in knee arthroscopy is a valid, useful, and accessible tool. The system provides standardized training for residents. These findings support the surgical simulation practice in a safe environment while providing feedback that should lead, we hope, to an effective transfer of learning to real surgical situations.
